# Case Report: Levetiracetam causing acute liver failure complicating post-operative management in a neurosurgical patient

**DOI:** 10.12688/f1000research.18198.1

**Published:** 2019-02-15

**Authors:** Sharanya Jayashankar, Sunil Munakomi, Vignesh Sayeerajan, Prakash Kafle, Pramod Chaudhary, Jagdishchandra Thingujam, Deepak Poudel, Iype Cherian

**Affiliations:** 1 Department of Neurosurgery, Nobel Medical College and Teaching Hospital, Biratnagar, 0977, Nepal

**Keywords:** Levetiracetam, anti-epileptics, hepatotoxicity

## Abstract

**Background:** Herein we report a rare case of acute liver failure due to levetiracetam, which has been considered to have an excellent safety profile with minimal hepatic side effects.

**Case presentation:** A 55-year-old male patient presenting with sudden onset dizziness, slurring of speech and headache was operated for posterior fossa cerebellar hematoma. His post-surgical period was complicated by development of icterus with elevation of liver enzymes. After ruling out common inciting factors, it was decided to stop levetiracetam which was given prophylactically for preventing seizures owing to presence of external ventricular drain. From the next day patient had dramatic improvement in liver functions and sensorium.

**Conclusions:** We would like to highlight this side effect that is potentially life threatening, though rare, of levetiracetam, which is very commonly used in today’s practice and fast superseding all other time-tested antiepileptics.

## Introduction

Seizure complicates major subsets of patients with stroke, and newer anti-epileptics are being favored in many clinical settings for seizure prophylaxis due to their good safety profile ^1^. Levetiracetam has become one of the most commonly used antiepileptic in current practice for treatment as well as prophylaxis against seizures.

Drug-induced liver injury owing to antiepileptic drugs (AED) is well recognized ^2^. It has been reported to occur more commonly with phenytoin and carbamazepine, and very rarely with valproate ^2^. The consequences of such an injury can be alarming, resulting in harbingering death or the need for liver transplantation. Therefore, newer AED with minimal or no hepatic metabolism are being favored as first line drugs ^3^.

Herein, we report a case of acute liver injury following levetiracetam usage in a post-operative patient of intracerebellar hemorrhage at our Neurosurgery Intensive Care Unit. We implicate a rare but life-threatening effect of a very commonly used anti-epileptic drug. There have been only few case reports of acute liver failure following use of levetiracetam and none were in post-operative neurosurgery cases ^4– 6^.

## Case report

A 55-year-old male patient from a remote village in Biratnagar presented to our emergency department with complaints of sudden onset dizziness, slurring of speech and headache. He was a known hypertensive but not on regular medication or regular follow-up. Neurological examination revealed Glasgow Coma Scale (GCS) of eye opening 4; Verbal 5; and Motor 5, on admission with his bilateral pupils equal and reactive to light. He had no focal neurological deficits or features of meningeal irritation. An emergent Computerized Tomography scan of the head showed features suggestive of cerebellar bleed with fourth ventricle compression with herniation and ventricular extension. While arranging for cerebral angiography, there was a sudden fall in GCS to E1V1M3, and thereby the patient underwent emergency suboccipital craniectomy with evacuation of cerebellar bleed with placement of external ventricular drain.

The patient’s post-operative medications included ceftriaxone (1gram intravenous every 8 ^th^ hourly), prophylactic levetiracetam (500 milligram intravenous every 12 ^th^ hourly), Pantoprazole (40 milligram intravenous every 12 ^th^ hourly), amlodipine (5 milligram via nasogastric tube 12 hourly), Losartan (50 milligram 12 hourly via Nasogastric tube), and Metoprolol (50 milligram via nasogastric tube 12 hourly). His immediate post-operative GCS improved to E3VtM6.

**Figure 1.**
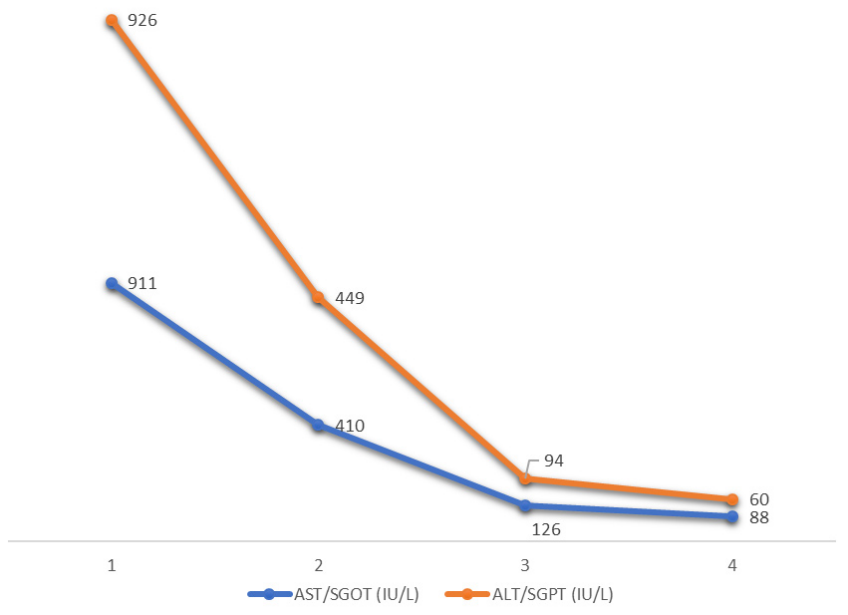
Changes in liver enzymes subsequent to stoppage of levetiracetam over a period of days (x-axis).

**Figure 2.**
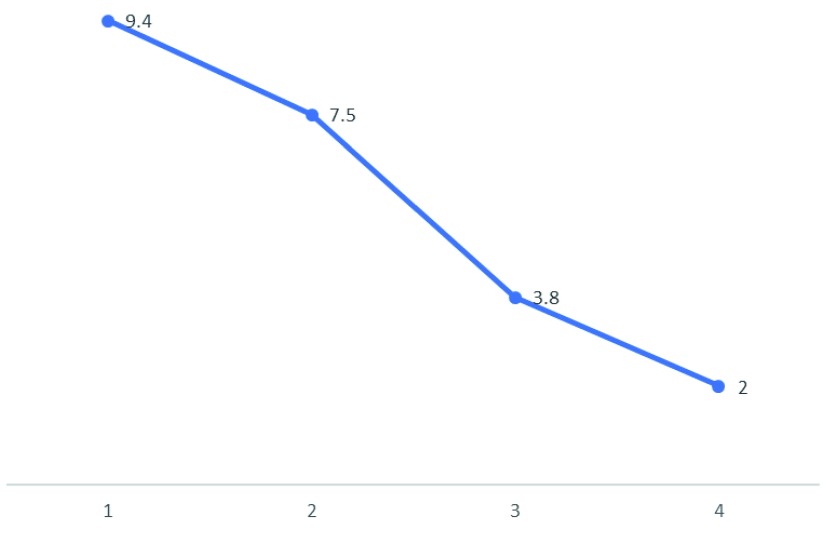
Changes in the bilirubin (mg/dl) subsequent to stoppage of levetiracetam over a period of days (x-axis).

However, on 3 ^rd^ post-operative day, the GCS fell to E1VTM4. Repeat CT head was uneventful. The patient was noted to have gross icterus and his liver function test revealed total bilirubin of 9.4 mg/dl (normal, 0.1mg/dl), direct 2.0 mg/dl (normal, 0-0.35mg/dl); aspartate aminotransaminase/serum glutamic-oxaloacetic transaminase (AST/SGOT) of 911 IU/L; (normal, 10–40 IU/L); alanine aminotransferase/serum glutamic-pyruvic transaminase (ALT/SGPT)of 926 IU/L (normal, 10–40 IU/L); alkaline phosphatase (ALP) of 298; (normal, 40–112 U/L); International Normalized ratio (INR) of 1.09 (normal, <1.1). Complete blood counts were done to rule out sepsis and were normal. Ultrasound of the abdomen and peripheral smear (for identifying features of obstructive jaundice as well as portal hypertension and ruling out hemolysis for raised bilirubin respectively) were normal. So, a possibility of drug induced liver injury causing acute hepatic failure was considered. Since none of the drugs prescribed were commonly implicated to have hepatotoxic effects, we considered the possibility of levetiracetam following a thorough literature search and hence stopped the drug. We also prescribed prophylactic hepatic encephalopathy regimen with strict monitoring of urine output, GCS, watching for seizures and features of upper gastrointestinal bleed. From the second day of stoppage of the drug, repeat laboratory tests showed gradual improvement in liver functions ( Figure 1 and Figure 2) paralleling clinical improvement.

Although restarting the patient with the same drug and aided with liver biopsy would be more diagnostic, in our case, the patient’s hepatic function rapidly normalized following stoppage of only levetiracetam from our prescribed drug lists. Therefore, we sufficiently concluded that levetiracetam caused the hepatotoxicity. Though rare, we would like to stress upon the importance of keeping this rare but life-threatening complications of levetiracetam in mind, as it can have profound effect on the timely and corrective management of the patient. There was no episode of recurrence of jaundice seen in the patient within the ensuing 3 weeks.

## Discussion

Seizures complicate up to 20% of cases with spontaneous intracerebral hemorrhage ^7^. There is no high level of evidence favoring the use of a specific AED ^7^. Levetiracetam is one of the most commonly used AED in current clinical practice due to its relatively good drug safety profile, and most adverse effects mentioned are usually mild to moderate in intensity ^8^. Levetiracetam does not inhibit or induce hepatic enzymes and most of it is eliminated unchanged by the kidneys. Thus, because it is minimally protein bound and lacks metabolism by the liver, the risk of hepatotoxicity is low. Thus, levetiracetam has a wide safety margin ^8^.

However, while reviewing literature, we found a few case reports citing hepatotoxicity with levetiracetam usage ^4– 6^. As per the current recommendations by the Council for International Organization of Medical Sciences (CIOMS) for diagnosing drug induced liver injury, our case was of hepatocellular variant ^9^. However, there is no consensus or diagnostic modality in correctly determining the implicated drugs, and thereby this has to be relied solely on the basis of empirical decision as to discontinue or modify drugs during such a scenario ^9^.

Tan *et al.* reported incidence of fulminant liver failure owing to levetiracetam ^4^. Syed and Adams also reported a case of liver failure following prophylactic levetiracetam usage in a patient with head injury ^5^. Sethi *et al.* reported a post-traumatic head injury patient who developed asymptomatic elevation of hepatic enzymes following levetiracetam usage ^6^.

## Conclusion

Though safe and free of major side effects when comparing to older AED, it is however prudent to note that there are reports of liver injury following levetiracetam, ranging from asymptomatic elevation of transaminases to fulminant hepatic failure. Though routine liver or renal function monitoring may not be needed, it is advisable to keep the patient informed of such possible side effects with the use of newer AED like levetiracetam.

## Consent

Since the patient was not fully conscious and alert enough to understand the concept of signing consent (since he was in a rehabilitation phase following intracranial hemorrhage), written informed consent for the publication of the relevant clinical and radiological data was obtained from the patient’s wife.

## Data availability

All data underlying the results are available as part of the article and no additional source data are required.
