# Supplementary material for: Case Report: Levetiracetam causing acute liver failure complicating post-operative management in a neurosurgical patient
Source: F1000Res. 2019 Feb 15;8:187. [Version 1] doi: 10.12688/f1000research.18198.1 (PMC6446496; doi:10.12688/f1000research.18198.1)
Supplement: Peer review history

# Peer review: Reviewer response for version 1

**DOI:** 10.5256/f1000research.19906.r46049

**Published:** 2019-04-01

**Authors:** Pathiyil Ravi Shankar

**Affiliations:** 1 Department of Pharmacology, Health City University, Gros Islet, Saint Lucia

My specific comments to further strengthen the manuscript are shown below. 

 Abstract Section:

 Case presentation subsection: Line 5, the word ‘inciting’ can be replaced by a more suitable word.

 Conclusions subsection: Line 1, ‘adverse effect’ may be a more appropriate term that ‘side effect’.

 Key words: More key words can be added. Key words can be arranged alphabetically.

 Page 2, column 1, Case report section, paragraph 1, Line 9: Do the authors mean emergency instead of emergent?

 The quality of written English is generally good but corrections may be required in a few places.

 I agree with the other reviewer that the authors can carry out a causality, severity and preventability assessment of the adverse drug reaction.     

 Also, information about the medicines used preoperatively and during anesthesia will be helpful as suggested by the other reviewer.

 Does the institution have a functioning pharmacovigilance center? Was the adverse drug reaction reported to the center?

I have read this submission. I believe that I have an appropriate level of expertise to confirm that it is of an acceptable scientific standard.

# Peer review: Reviewer response for version 1

**DOI:** 10.5256/f1000research.19906.r46050

**Published:** 2019-03-25

**Authors:** Subish Palaian

**Affiliations:** 1 Department of Pharmacy Practice, College of Pharmacy, Gulf Medical University, Ajman, United Arab Emirates

**General comments: **It is a timely report representing an important area of clinical practice and hence deserves indexing.

**Specific comments: **

- Authors must mention these points in the case report section:

Whether this patient had a history of liver problems in the recent past?

 Any previous history of allergy to any other drugs?

 Was there use of potentially hepatotoxic general anaesthetic agent?

 Is he taking any other medications at the time of admission?

 Is the patient an alcoholic?

 Any other predisposing factors that could have potentially caused/augmented this adverse drug reaction?  

- Causality assessment: It will be nice for the authors to perform a ‘Causality assessment’ and grade the Causal relationship between the drug and the event. I recommend using Naranjo scale.
- Severity assessment: The severity assessment gives an opportunity for the authors to categorize the adverse drug reaction based on its severity. Authors could sue the ‘Hartwig Scale’ for this purpose.
- In ‘Case report’ Section, …remote village in Biratnagar, Nepal. (add ‘Nepal’) 
- ‘Discussion section’ should emphasize on the management pattern patient (in relation to the adverse drug reactions) highlighting any supportive treatment provided in this patient. This could be even mentioned partly under the ‘Case report’ section.

I have read this submission. I believe that I have an appropriate level of expertise to confirm that it is of an acceptable scientific standard.
